# Immunohistochemical Analysis of the Mechanistic Target of Rapamycin and Hypoxia Signalling Pathways in Basal Cell Carcinoma and Trichoepithelioma

**DOI:** 10.1371/journal.pone.0106427

**Published:** 2014-09-02

**Authors:** Tjinta Brinkhuizen, Chantal A. H. Weijzen, Jonathan Eben, Monique R. Thissen, Ariënne M. van Marion, Björn G. Lohman, Véronique J. L. Winnepenninckx, Patty J. Nelemans, Maurice A. M. van Steensel

**Affiliations:** 1 Department of Dermatology, Maastricht University Medical Centre, Maastricht, the Netherlands; 2 Department of Pathology, Maastricht University Medical Centre, Maastricht, the Netherlands; 3 Department of Epidemiology, Maastricht University Medical Centre, Maastricht, the Netherlands; 4 Department of Clinical Genetics, Maastricht University Medical Centre, Maastricht, the Netherlands; 5 GROW, School for Oncology and Developmental Biology, Maastricht University Medical Centre, Maastricht, the Netherlands; 6 Department of Pathology, VieCuri medical Centre, Venlo, the Netherlands; 7 Department of Pathology, Laurentius Hospital, Roermond, the Netherlands; 8 Institute of Medical Biology, Immunos, Singapore, Singapore; University of Dundee, United Kingdom

## Abstract

**Background:**

Basal cell carcinoma (BCC) is the most common cancer in Caucasians. Trichoepithelioma (TE) is a benign neoplasm that strongly resembles BCC. Both are hair follicle (HF) tumours. HFs are hypoxic microenvironments, therefore we hypothesized that hypoxia-induced signalling pathways could be involved in BCC and TE as they are in other human malignancies. Hypoxia-inducible factor 1 (HIF1) and mechanistic/mammalian target of rapamycin (mTOR) are key players in these pathways.

**Objectives:**

To determine whether HIF1/mTOR signalling is involved in BCC and TE.

**Methods:**

We used immunohistochemical staining of formalin-fixed paraffin-embedded BCC (*n* = 45) and TE (*n* = 35) samples to assess activity of HIF1, mTORC1 and their most important target genes. The percentage positive tumour cells was assessed manually in a semi-quantitative manner and categorized (0%, <30%, 30–80% and >80%).

**Results:**

Among 45 BCC and 35 TE examined, expression levels were respectively 81% and 57% (BNIP3), 73% and 75% (CAIX), 79% and 86% (GLUT1), 50% and 19% (HIF1α), 89% and 88% (pAKT), 55% and 61% (pS6), 15% and 25% (pMTOR), 44% and 63% (PHD2) and 44% and 49% (VEGF-A). CAIX, Glut1 and PHD2 expression levels were significantly higher in TE when only samples with at least 80% expression were included.

**Conclusions:**

HIF and mTORC1 signalling seems active in both BCC and TE. There are no appreciable differences between the two with respect to pathway activity. At this moment immunohistochemical analyses of HIF, mTORC1 and their target genes does not provide a reliable diagnostic tool for the discrimination of BCC and TE.

## Introduction

**Figure 1:**
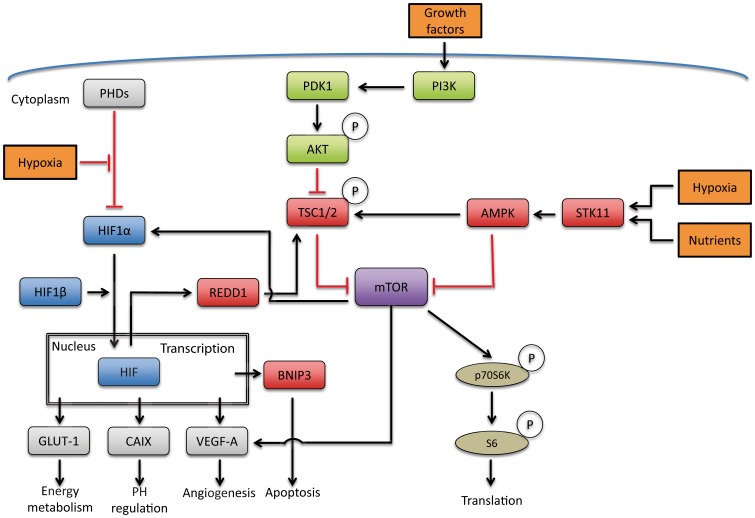
Simplified depiction of pathways affected by hypoxia. Under hypoxic conditions the expression of prolyl hydroxylase domain proteins (PHDs) is reduced leading to an induction of hypoxia inducible factor 1α (HIF1α) expression, which becomes stable and active as a transcription factor, together with hypoxia inducible factor 1β (HIF1β). HIF1 activation regulates the expression of several target genes whose products address the needs of oxygen starved cells, such as vascular endothelial growth factor (VEGF-A), BCL-2/adenovirus E1B 19 kDa-interacting protein 3 (BNIP3), glucose transporter 1 (GLUT1) and glycolytic enzymes such as carbonic anhydrase IX (CAIX). Hypoxia also influences mechanistic/mammalian target of rapamycin complex 1 (mTORC1) signalling mainly mediated through hypoxic activation of the TSC1-TSC2 complex by REDD1. First, phosphatidylinositol 3 kinase (PI(3)K) and protein kinase B (AKT) have been implicated in the activation of the mTOR protein kinase. One critical target of AKT that regulates mTOR is the tumour suppressor protein, tuberin (TSC2). Tuberin negatively regulates mTOR signalling, and AKT activation circumvents this inhibition. Constitutive mTOR signalling positively stimulates S6 kinase (S6K), a downstream effector of mTOR pathway, which mainly drives cell growth and proliferation. Also, mTOR enhances the protein levels of HIF and consequently enhances the expression of HIF target genes. Second, under conditions of hypoxia, intracellular ATP levels drop and AMP levels rise. AMP directly binds to a subunit of AMP activated protein kinase (AMPK), which is then phosphorylated by serine/threonine protein kinase 11 (STK11/LKB1). Elevated concentrations of AMPK can cause a complete inhibition of mTOR (mTORC1) activity without affecting PI(3)K-AKT signalling.

Basal cell carcinoma (BCC) is the most common cancer in Caucasians. Its incidence is increasing by 3–10% annually, leading to a lifetime BCC risk in Caucasians of approximately 1 in 5–6. [1] Sun-exposed body sites like the head and neck are most commonly affected. [1] Histologically, BCC can be classified into three main subtypes: superficial, nodular and infiltrative. [2] Youssef et al. were the first to show that BCC are epithelial tumours originating from progenitor cells of the interfollicular epidermis (IFE) and the upper infundibulum. [3] Later evidence shows that BCC can also arise from the hair follicle (HF) bulge. [4] Trichoepithelioma (TE) is a similar, though benign epithelial tumour which expresses markers that are also present in the outer root sheath of the HF [5], [6], which suggests that TE originate from the HF as well. Two distinct subtypes of TE are recognized: the classic and the desmoplastic type. [6] Some TE can mimic BCC macroscopically, [6] making it difficult to differentiate between both tumours and this dilemma can extend to the microscopic level. Histologically, both BCC and TE are characterised by dermal nests of basaloid cells. TE differs from BCC by its absence of peripheral palisading of basaloid keratinocytes, necrosis, retraction artefact, mitotic activity and peritumoral mucin deposition. Also, TE rarely ulcerates. BCC conversely is usually not associated with formation of horn cysts and papillary mesenchymal bodies. However histological distinction is predominantly based on the higher degree of follicular differentiation as found in TE. [6] Differentiation between these two HF derived tumours is important because of their distinct biologic behaviour and therapeutic approach. Decreased oxygen levels are a common feature in many tumours [7], including cutaneous squamous cell carcinoma and melanoma. [8] While the human dermis is well-oxygenated and the epidermis is modestly hypoxic, the HF is considered as a moderately to severely hypoxic microenvironment. [9] A primary mediator of hypoxia-induced gene expression in human HFs is the hypoxia-inducible transcription factor 1 (HIF1). [10] HIF1 is activated in several tumour types, [11] and is mainly negatively regulated by prolyl hydroxylase 2 (PHD2); the key oxygen sensor. [12] In hypoxic conditions, HIF1α regulates the expression of important proteins including vascular endothelial growth factor (VEGF-A), glucose transporter 1 (GLUT1), catalytic enzymes such as carbonic anhydrase IX (CAIX) and pro-apoptotic BCL-2/adenovirus E1B 19 kDa-interacting protein 3 (BNIP3). VEGF-A is suggested as a key driver in the angiogenic response and is overexpressed in most solid cancer. As a consequence, inhibition of VEGF-A can suppress tumour growth. [13] GLUT1 is responsible for glucose uptake and the expression of GLUT1 increases under hypoxic conditions inducing glycolysis. [14] Tumours generally have high rates of glycolysis (the Warburg effect). [15] CAIX is involved in several regulatory process that are beneficial for tumour survival, the most important is pH regulation. Aggressive tumour behaviour and poor patient outcome is associated with high CAIX expression levels. [16] Also BNIP3 is associated with poor prognosis in some cancers, promoting apoptosis and even autophagy. [17] Hence, all of these genes play critical roles in tumour growth. [18] Of note, they can also be induced by mechanistic/mammalian target of rapamycin (mTORC1) signalling [19] which is activated in several tumour types. [20] Also, mTORC1 enhances the protein levels of HIF via HIF1α and consequently enhances the expression of HIF target genes. [21] Dodd et al additionally showed that mTORC1 mediates VEGF-A expression via both HIF1α dependent and independent mechanisms. [19] Conversely, hypoxia promotes tuberous sclerosis complex 2 (TSC2) via ‘regulated in development and DNA damage responses 1′ (REDD1) that leads to inhibition of mTORC1 and reveals a feedback mechanism where HIF can turn off mTOR complex 1 (mTORC1). [22] mTOR, as part of mTORC1, plays an integral role in the coordination of cell growth and division in response to growth factors, nutrients and the energy status of the cell (Figure 1.)). [22] [23] Human tumours are characterised by an heterogeneous microenvironment that differs from normal tissue with respect to nutrient supply, pH and oxygenation. As discussed, deficiencies in oxygenation (hypoxia) is strongly associated with tumour development, growth, metastasis and poor response to therapy. HIF and mTORC1 signalling are crucial in here, either directly or indirectly acting on the hypoxia response. [23]

Based on the hypoxia affecting the HF and the known role of both HIF and mTORC1 in tumour growth, we have previously hypothesized that hypoxia response pathways are involved in the pathogenesis of HF tumours. [10] If HIF and mTORC1 pathways do contribute to the growth of HF tumours, new opportunities for targeted therapy and diagnostics could emerge.

Therefore, we decided to examine BCC as this is the most prevalent HF tumour and included the very similar TE. [9], [10], [24] We analysed the expression of the most important target genes in the hypoxia response using immunohistochemical stains of both BCC and TE. To the best of our knowledge, this is the first large series of BCC and TE to date to be analysed for HIF1 and mTORC1 activity. Our results suggest that HIF1/mTORC1 mediated signalling is active in both tumour types.

## Materials and Methods

### Tissue samples

Collection, storage and use of all tissues and patient data were performed in agreement with the "Code for Proper Secondary Use of Human Tissue in the Netherlands". We randomly selected 45 BCC and 80 TE haematoxylin-eosin stain (HE) slides from the Pathologic Anatomical National Automated Archive of the Department of Pathology in the Maastricht University Medical Centre (MUMC). A combination of search terms was used to retrieve reports of the specific histological diagnosis. Based on the morphological classification by Rippey, BCC were subcategorised into superficial, nodular and infiltrative during routine histological diagnostic examination. [2] All TE HE slides were reviewed by three independent investigators (*i.e.* a dermato-pathologist (AM), a general pathologist (BL) and a dermatologist (MT)), because the histopathologic diagnosis of TE can present considerably more difficulties than that of BCC. The 80 slides were reviewed based on the presence or absence of the following characteristics: tumour-stroma cleft formation, ulceration, epithelial primitive structures, small keratinous cysts, inflammatory response, mitosis, necrotic tumour cells, papillary mesenchymal bodies, stromal oedema and peritumoral mucin production. [5] Eventually, 35 TE were found to unambiguously fit the criteria for classic TE and these were used for the analysis, together with 45 BCC. All of the used samples and corresponding data were de-linked and anonymised. Usage of tissue samples was approved by the Maastricht Pathology Tissue Collection (MPTC) scientific committee (MPTC 2009-05).

### Immunohistochemistry

Formalin fixed and paraffin embedded (FFPE) biopsies as well as excision specimens were used. Four-micrometre sections were cut and stained with primary antibodies, listed in table 1. Hif1α, CAIX, Glut-1 and VEGF-A stains were performed on a Dako autostainer system with use of a pre-treatment module using EnVision FLEX Target Retrieval Solution, High pH (Dako, Heverlee, Belgium). The antibodies were applied for 20 minutes at room temperature. For HIF1α, the slides were additionally incubated with Envision Flex Mouse Linker to amplify the signal. The Dako Envision Flex kit (K8002) was used for secondary detection.

**Table 1:** Antibodies used for immunohistochemical analysis.

Antibody	Dilution	Producer
BNIP3	1∶800	Sigma Aldrich, St. Louis, U.S.A.
CAIX	1∶1000	Novus Biologicals, Littleton, U.S.A.
GLUT1	1∶200	Thermo Scientific, Landsmeer, the Netherlands
HIF1α	1∶50	BD Transduction Laboratories, Breda, the Netherlands
pAKT	1∶25	Cell Signaling technology, Beverly, MA, U.S.A.
pS6	1∶50	Cell Signaling technology, Beverly, MA, U.S.A.
pMTOR2448	1∶50	Cell Signaling technology, Beverly, MA, U.S.A.
PHD2	1∶250	Novus Biologicals, Littleton, US
VEGF-A	1∶200	Thermo Scientific, Landsmeer, the Netherlands

BCL2/adenovirus E1B 19 kDa protein-interacting protein 3 (BNIP3), carbonic anhydrase IX (CAIX), glucose transporter member 1 (GLUT1), hypoxia-inducible factor 1-alpha (HIF1α), phosphorylated -protein kinase B (pAKT), phosphorylated-S6 (pS6), phosphorylated-mechanistic target of Rapamycin (pMTOR), prolyl hydroxylase domain protein 2 (PHD2), vascular endothelial growth factor (VEGF-A).

For BNIP3, PHD2, pS6, pMTOR2448 and pAKT, slides were deparaffinised in xylene, rehydrated and incubated in 0·3% (PHD2, pMTOR2448) or 3% (BNIP3, pS6) hydrogen peroxide (H_2_O_2_) in methanol for 30 minutes to inactivate endogenous peroxidase activity. Antigen retrieval was performed by microwave treatment at 90 W for 10 minutes in 10 mM citrate buffer (pH 6) and non-specific protein binding was blocked using 3% bovine-serum-albumin (BSA). The sections were incubated for 1 hour at room temperature (BNIP3, PHD2) or overnight at 4°C (pS6, pMTOR2448, pAKT). For secondary detection an Envision detection system was used and bound antibody was visualized by using 3,3-diaminobenzidine (DAB) for 10 minutes. After secondary detection, all sections were counterstained with Gill II haematoxylin, dehydrated and coverslipped. In all reactions, appropriate positive and negative controls were included and always showed the expected positive resp. negative results.

### Interpretation of the stains

A trained medical student of the Department of Dermatology, MUMC (CW) and an experienced resident of the Department of Pathology, MUMC (JE) examined all sections. Both were blinded for patient details. Any discrepancy between the observers was discussed and resolved by consensus. For all tumours, at least four randomly chosen high-power fields (magnification 200x) per slide were assessed to determine the percentage positive tumour cells. The percentage of cell staining was scored as 0 (no staining), 1 (<30% staining), 2 (30–80% staining) and 3 (>80% staining)[25]. For all assessments, the HF was used as internal standard and considered as 100% positive.

### Statistical analysis

Statistical analyses were carried out using SPSS version 20.0 software (SPSS, Chicago, IL, USA). Descriptive data were presented as absolute numbers and percentages for categorical data and as means with standard deviations for continuous data. The Chi-square test for independent proportions was performed to evaluate the differences and similarities in expression of CAIX, BNIP3, GLUT1, HIF1α, pAKT, PHD2, pMTORC1, pS6 and VEGF-A between BCC and TE specimens. Correlations among the different stainings were assessed by Spearman's correlation coefficient. P<0·05 was considered to be statistically significant.

## Results

### Sample characteristics

Forty-five BCC (superficial n = 14, nodular n = 24, and infiltrative n = 7) were included. Among the 80 TE cases selected, 35 were unanimously found to be classic type TE. Distribution of patient and tumour characteristics within the TE and BCC group are equal and listed in table 2.

**Table 2:** Tumour characteristics.

	Trichoepithelioma	Basal cell carcinoma
Number tumours	35	45
Biopsy/excision*	19/16 (54.3/45.7)	0/45 (0.0/100.0)
Gender (m/f)*	16/21 (43.2/56.8)	24/21 (53.3/46.7)
Mean patient age in years	58.8	68.6
Tumour localisation*		
Head	28 (80.0)	18 (40.0)
Nek	3 (8.6)	4 (8.9)
Trunk	3 (8.6)	15 (33.3)
Arm	0.0 (0.0)	2 (4.4)
Leg	1 (2.9)	6 (13.3)
TE Subtype*		
Classic TE	35 (100.0)	
Desmoplastic TE	0 (0.0)	
BCC Subtype*		
Superficial BCC		14 (31.1)
Nodular BCC		24 (53.3)
Infiltrative BCC		7 (15.6)

*Date are presented as n (%).

### Hypoxia target staining patterns in basal cell carcinoma and trichoepithelioma

**Figure 2:**
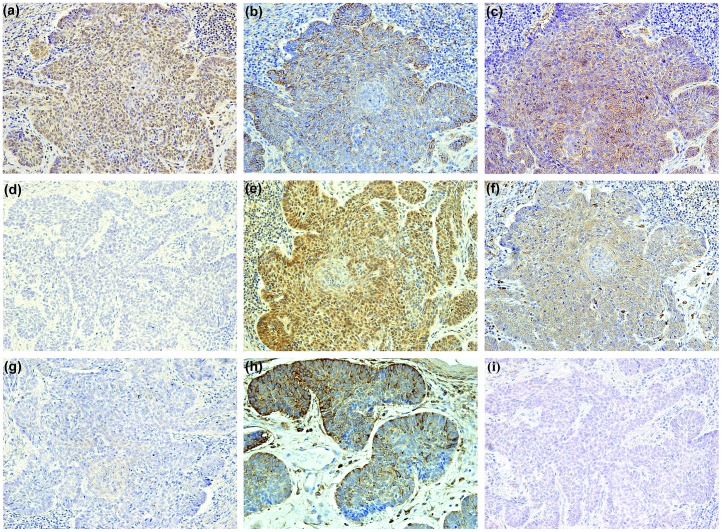
Immunohistochemical stains for hypoxia inducible factor 1α (HIF1α), phosphorylated mechanistic/mammalian target of rapamycin (pmTOR) and their target genes in basal cell carcinoma (BCC). BCL-2/adenovirus E1B 19 kDa-interacting protein 3 (BNIP3) (**a**); carbonic anhydrase IX (CAIX) (**b**); glucose transporter 1 (GLUT1) (**c**); HIF1α (**d**); phosphorylated protein kinase B (pAKT) (**e**);phosphorylated ribosomal protein S6 (pS6) (**f**); pmTOR (**g**); prolyl hydroxylase domain protein 2 (PHD2) (**h**); vascular endothelial growth factor (VEGF-A) (**i**). Original magnification: (a–i) x 200.

**Figure 3:**
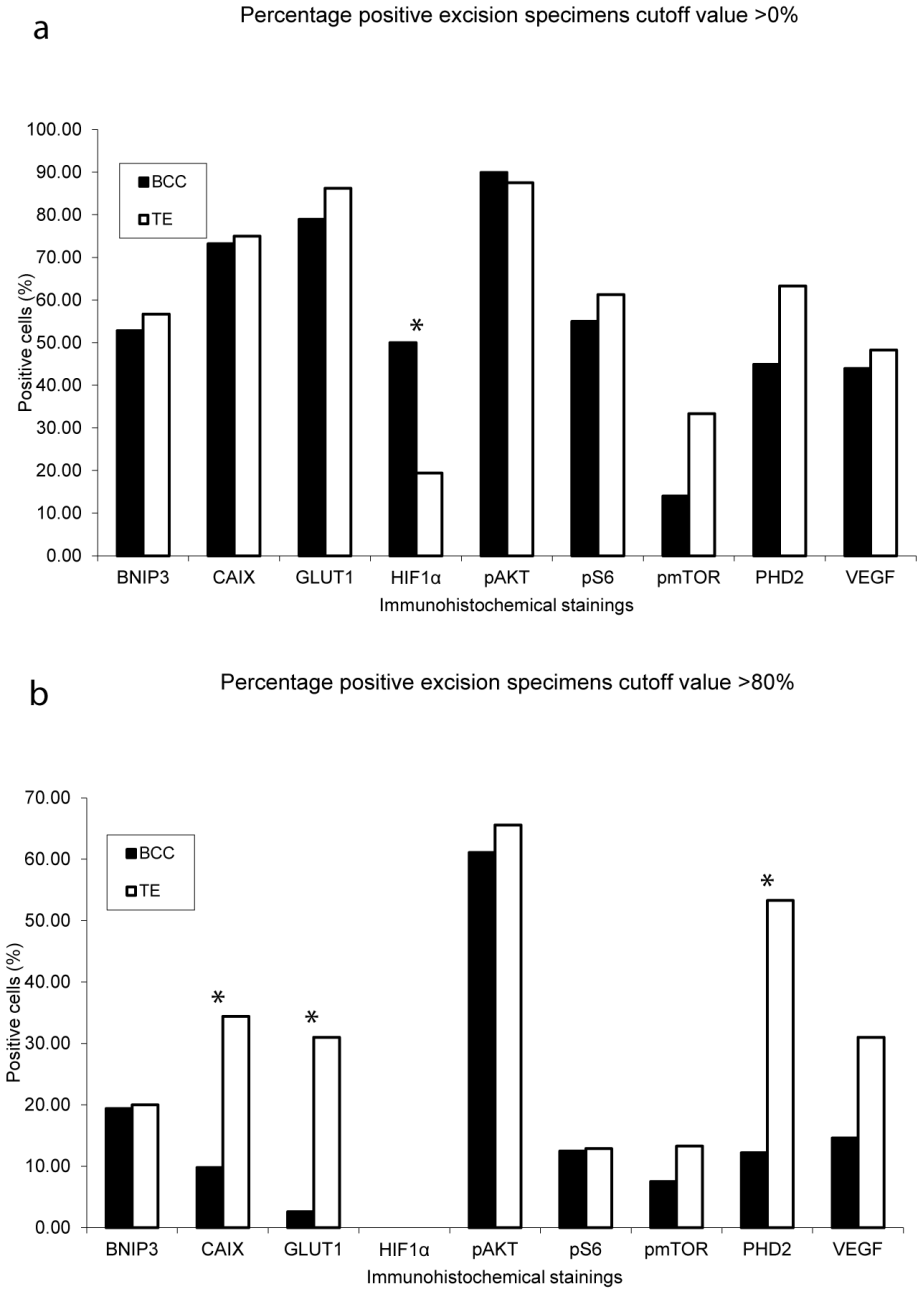
Percentage of positive specimens between basal cell carcinoma and trichoepithelioma. Panel a represents all tissue samples being either positive or negative. In panel b the same results are shown, however here a cut off value of 80% of the tumour cells being positive was used. * (P<0·05), basal cell carcinoma (BCC); trichoepithelioma (TE).

We investigated the activation status of hypoxia and mTORC1 signalling cascades by immunohistochemical analysis of BCC and TE. Immunohistochemical staining of BNIP3, GLUT1, CAIX, VEGF-A and PHD2 was observed in the suprabasal portion of the overlying epidermis of the BCC and TE tumour islands and in the internal control, the HF. Immunohistochemical staining for HIF1α was negative in epidermis overlying the tumour islands, while the HF showed weak scattered positive nuclear expression. VEGF-A showed expression in the endothelial cells of blood vessels. An overview of all stains is presented in figure 2 and 3.

**Figure 4:**
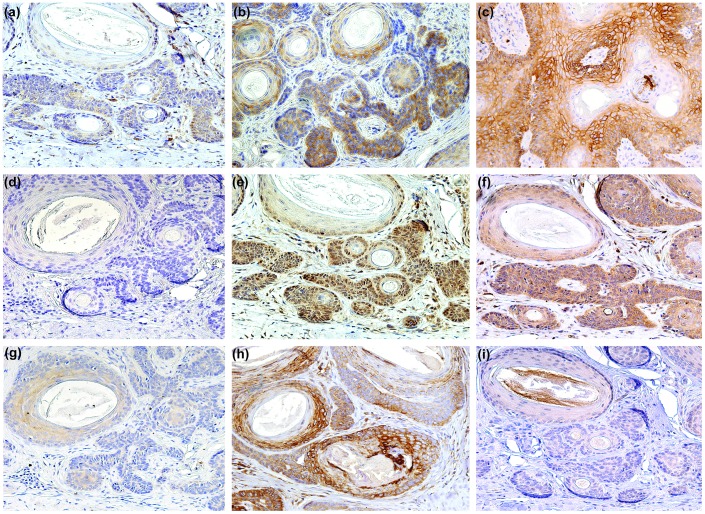
Immunohistochemical staining for hypoxia inducible factor 1α (HIF1α) and phosphorylated mechanistic/mammalian target of rapamycin (pmTOR) and their target genes in trichoepithelioma (TE). BCL-2/adenovirus E1B 19 kDa-interacting protein 3 (BNIP3) (**a**); carbonic anhydrase IX (CAIX) (**b**); glucose transporter 1 (GLUT1) (**c**); HIF1α (**d**); phosphorylated protein kinase B (pAKT) (**e**); phosphorylated ribosomal protein S6 (pS6) (**f**); p-mTOR (**g**); prolyl hydroxylase domain protein 2 (PHD2) (**h**); vascular endothelial growth factor (VEGF-A)(**i**). Original magnification: (a–i) x 200.

Overall, about half of all BCC and TE indicate active hypoxia signalling, however the BCC specimens tended to have less CAIX, GLUT1, and PHD2 expression than TEs (Fig. 4a). In TE high expression levels (>80%) of PHD2, GLUT1 and CAIX were observed in 53·3%, 31·0% and 34·4%, respectively (Fig. 4b). Conversely, HIF1α was more often positive in BCC nuclei than in TE (50·0% vs 19·4% (P<0.05)). The downstream effectors BNIP3 and VEGF-A showed no significantly different expression levels. When examining different BCC subtypes, nodular BCC harboured more GLUT1 and PHD2 expression compared to superficial and infiltrative BCC with P-values of respectively P = 0·032 and P = 0·17. An overview of the expression in BCC and TE is presented in table 3.

**Table 3:** The expression levels and staining intensity of BNIP3, CAIX, GLUT1, Hif1α, pAKT, PHD2, pmTOR, pS6 and VEGF-A in hair follicle tumours.

			Percentage positive cells				Subgroup analysis (P-value)
Staining	Group	N	0, n (%)	<30%, n (%)	30–80%, n (%)	>80%, n (%)	
BNIP3	BCC	36	17	1	11	7	0,670
			(47.2)	(2.8)	(30.6)	(19.4)	
	TE	30	13	3	8	6	
			(43.3)	(10.0)	(26.7)	(20.0)	
CAIX	BCC	41	11	10	16	4	0,048
			(26.8)	(24.4)	(39.0)	(9.8)	
	TE	32	8	3	10	11	
			(25.0)	(9.4)	(31.3)	(34.4)	
GLUT1	BCC	38	8	16	13	1	0,007
			(21.1)	(42.1)	(34.2)	(2.6)	
	TE	29	4	12	4	9	
			(13.8)	(41.4)	(13.8)	(31.0)	
HIF1α	BCC	36	18	12	6	-	0,015
			(50.0)	(33.3)	(16.7)		
	TE	31	25	2	4	-	
			(80.6)	(6.5)	(12.9)		
pAKT	BCC	36	4	1	9	22	0,791
			(11.1)	(2.8)	(25.0)	(61.1)	
	TE	32	4	-	7	21	
			(12.5)		(21.9)	(65.6)	
PS6	BCC	40	18	9	8	5	0,935
			(45.0)	(22.5)	(20.0)	(12.5)	
	TE	31	12	7	8	4	
			(38.7)	(22.6)	(25.8)	(12.9)	
pMTOR	BCC	40	34	3	-	3	0,083
			(85.0)	(7.5)		(7.5)	
	TE	30	20	2	4	4	
			(66.7)	(6.7)	(13.3)	(13.3)	
PHD2	BCC	41	23	3	10	5	0,002
			(56.1)	(7.3)	(24.4)	(12.2)	
	TE	30	11	1	2	16	
			(36.7)	(3.3)	(6.7)	(53.3)	
VEGF-A	BCC	41	23	6	6	6	0,344
			(56.1)	(14.6)	(14.6)	(14.6)	
	TE	29	15	2	3	9	
			(51.7)	(6.9)	(10.3)	(31.0)	

BNIP3, BCL-2/adenovirus E1B 19 kDa-interacting protein 3; CAIX, carbonic anhydrase IX; GLUT1, glucose transporter 1; Hif1α, hypoxia inducible factor 1α; pAKT, phosphorylated protein kinase B; pS6, phosphorylated ribosomal protein S6, pMTOR, phosphorylated mechanistic target of rapamycin; PHD2, prolyl hydroxylase domain protein 2; VEGF-A, vascular endothelial growth factor; BCC, basal cell carcinoma; TE, trichoepithelioma. Expression levels were graded semiquantitatively as 0%, 1–30%, 30–80% or >80% positive tumour cells.

### mTORC1 target staining patterns in basal cell carcinoma and trichoepithelioma

An overview of all stains is shown in figure 2,3,4 and table 3. pAKT and pS6 were positive in the epidermis overlying the BCC and TE tumour islands and the HF, while immunohistochemical expression of phosphorylated mTORC1 at Ser-2448 (pmTOR) in the overlying epidermis was negative. The HF showed some weak and scattered pmTOR staining. In contrast, we found pAKT to be highly expressed in 88·9% of BCC and 87·5% in TEs (P = 0·791). As a downstream effector of pAKT, pmTOR exhibited positive intracytoplasmic and partly membranous expression in only 15·0% of the BCC, while 33·3% of the TE showed positive staining (P = 0·083). pS6 showed no significant difference between intracytoplasmic staining expression in BCC (55·0%) specimens and TE (63·3%) specimens (P = 0·935). No differences among the BCC subtypes could be detected.

### Spearman's correlation coefficients among HIF and mTORC1 signaling target genes

All stains were statistically analyzed for correlation of expression patterns revealing the following significant correlations. In BCC, significant correlations between CAIX and GLUT1 (r = 0·369, P = 0·024), CAIX and pS6 (r = 0·522, P = 0·001) and CAIX and PHD2 (r = 0·426, P = 0·007) were detected as well as between PHD2 and BNIP3 (r = 0·485, P = 0·003) and PHD2 and pS6 (r = 0·399, P = 0·012). Furthermore, the expression of VEGF-A correlated significantly with that of pMTOR (r = 0·342, P = 0·033). Moreover in TE, PHD2 was significantly correlated with CAIX (r = 0·438, P = 0·017) and GLUT1 (r = 0·544, P = 0·003). Lastly, pAKT expression correlates significantly with pS6 (r = 0·438, P = 0·015) and VEGF-A with pMTOR (r = 0·502, P = 0·07) in TE.

## Discussion

The HIF and mTORC1 signalling pathways play crucial roles in many malignancies. [23] To the best of our knowledge, we are the first to perform a systematic analysis of HIF and mTORC1 signalling in both BCC and TE. Although TE showed significantly more expression of CAIX, Glut1 and PHD2, this was only in the proportion of samples with high expression levels of at least 80% and thus not likely to reflect a genuine difference between the two tumour types. The correlations found between components of HIF signalling and the mTORC1 pathway are in line with known crosstalk between both pathways.[23] GLUT1, CAIX and BNIP3 are the most important readouts for HIF1α signalling [26]–[28] and our data show positivity of all three target genes in the majority of both BCC and TE. Positive VEGF-A staining patterns were found in the endothelial cells of blood vessels as well as in BCC/TE tumour cells. However, VEGF-A expression was not specifically tumour related, since some weak staining was also noted in the basal cell layer of epidermis adjacent to the tumours. These low and weak VEGF-A expression levels are in agreement with earlier studies in BCC and other benign and malignant skin tumours [29], [30] and might be explained by the low levels of mTORC1 expression found (see below), since recent evidence indicates that mTORC1 can drive VEGF-A expression. [19] It also fits with the low metastatic behaviour of BCC [29]. Accordingly, strong VEGF-A expression is observed in the more aggressive and potentially metastasizing squamous cell carcinoma and melanoma. [29], [31]

Furthermore, we found expression of PHD2 in 63·3% of TE and 43·9% of BCC specimens, whereas expression of HIF1α itself was low or even undetectable. Several *in vitro* studies have shown that PHD2 is transiently upregulated in a HIF-dependent manner under normoxic as well as mild hypoxic conditions, which could suggest that HIF might induce an autoinhibitory effect on its own activity. [32] Moreover, D'Angelo et al demonstrated that hypoxic upregulation of PHD2 acts as a feedback mechanism to stop hypoxic signalling in reoxygenated cells. [33] Hence, we postulate that the lack of HIF1α in BCC and TE specimens can be explained by the presence of PHD2.

Activity of the mTORC1 pathway was assessed by use of the upstream regulator pAKT and downstream target pS6. pAKT was positive in 88·9% of BCC and 87·5% of TE, while 55% of BCC and 61·3% of TE specimens were positive for pS6, supporting the presence of active mTOR signalling. The positive stains for pAKT and pS6 found in our study are consistent with the known activity of PI(3)K/AKT signalling in BCC. [34] In addition, strong expression of pAKT and pS6 has been observed in a variety of skin neoplasms including Bowen's disease, keratoacanthoma, squamous cell carcinoma and extramammary Paget's disease. [35], [36] However, despite positive staining of pAKT and pS6, hardly any mTORC1 phosphorylation at Ser-2448 was detected in both tumour types. This observation is consistent with two other studies reporting weak positivity of mTOR (Ser-2448) in only 7·7% of BCC [37] and 36% positivity among 85 epidermal tumours other than BCC. [36] Ser-2448 is the mTORC1phosphorylation site modified either directly by AKT or by the downstream target of mTORC1, p70S6 kinase, making it the most important marker for activation of mTOR. [38] However, the upregulation of up- and downstream target genes of the mTORC1 signalling cascade do suggest activation of downstream mTORC1 signalling components downstream, which could be attributed to PI(3)K- signalling. [39] In addition, it is known that multiple feedback loops exist, for example S6 kinase can dampen growth factor receptor signalling to PI3K. [40]

Overall the immunohistochemical analysis is consistent with activity of HIF1 and mTORC1 signalling in both BCC and TE, in addition to the known PI(3)K-AKT activity in BCC. [34] We also showed that the number of HIF1α positive cells was significantly higher in BCC than in TE, which might be explained by the fact that BCC tumour nests are generally larger than the TE tumour nests and consequently could be more hypoxic. Nodular BCC tend to have more GLUT1 and PHD2 expression when compared to superficial and infiltrative BCC, which could similarly be due to them being more hypoxic.

Advanced insights in molecular pathways active in cancer development have already resulted in the development of novel topical and systemic targeted therapies as a rational approach to the management of many (skin) cancers. [41] Our results suggest that it might be of interest to further explore the contribution of HIF1 and mTORC1 signalling to BCC and TE growth. Deeper insights into such signalling pathways might eventually result in the identification of novel targets for treatment. Finally a better understanding of alterations in gene expression could be used to develop better histological diagnostics, since at this moment immunohistochemical analysis of HIF, mTORC1 and their target genes does not provide a reliable diagnostic tool for the discrimination of BCC and TE.

## References

[1] Flohil SC, Seubring I, van Rossum MM, Coebergh JW, de Vries E, et al (2013) Trends in Basal cell carcinoma incidence rates: a 37-year Dutch observational study. J Invest Dermatol 133: 913–918. 23190883 10.1038/jid.2012.431

[2] Rippey JJ (1998) Why classify basal cell carcinomas? Histopathology 32: 393–398. 9639112 10.1046/j.1365-2559.1998.00431.x

[3] Youssef KK, Van Keymeulen A, Lapouge G, Beck B, Michaux C, et al (2010) Identification of the cell lineage at the origin of basal cell carcinoma. Nat Cell Biol 12: 299–305. 20154679 10.1038/ncb2031

[4] Kasper M, Jaks V, Hohl D, Toftgard R (2012) Basal cell carcinoma - molecular biology and potential new therapies. J Clin Invest 122: 455–463. 22293184 10.1172/JCI58779 PMC3266783

[5] Arits AH, Van Marion AM, Lohman BG, Thissen MR, Steijlen PM, et al (2011) Differentiation between basal cell carcinoma and trichoepithelioma by immunohistochemical staining of the androgen receptor: an overview. Eur J Dermatol 21: 870–873. 21865121 10.1684/ejd.2011.1504

[6] Weedon D (2009) Skin Pathology. Tokyo: Churchill Livingstone.

[7] Yang Y, Sun M, Wang L, Jiao B (2013) HIFs, angiogenesis, and cancer. J Cell Biochem 114: 967–974. 23225225 10.1002/jcb.24438

[8] Nys K, Maes H, Dudek AM, Agostinis P (2011) Uncovering the role of hypoxia inducible factor-1alpha in skin carcinogenesis. Biochim Biophys Acta 1816: 1–12. 21338656 10.1016/j.bbcan.2011.02.001

[9] Evans SM, Schrlau AE, Chalian AA, Zhang P, Koch CJ (2006) Oxygen levels in normal and previously irradiated human skin as assessed by EF5 binding. J Invest Dermatol 126: 2596–2606. 16810299 10.1038/sj.jid.5700451

[10] van Steensel MA, van Geel M, Badeloe S, Poblete-Gutierrez P, Frank J (2009) Molecular pathways involved in hair follicle tumor formation: all about mammalian target of rapamycin? Exp Dermatol 18: 185–191. 19146581 10.1111/j.1600-0625.2008.00808.x

[11] Ruan K, Song G, Ouyang G (2009) Role of hypoxia in the hallmarks of human cancer. J Cell Biochem 107: 1053–1062. 19479945 10.1002/jcb.22214

[12] Chan DA, Giaccia AJ (2010) PHD2 in tumour angiogenesis. Br J Cancer 103: 1–5. 20461086 10.1038/sj.bjc.6605682 PMC2905285

[13] Vasudev NS, Reynolds AR (2014) Anti-angiogenic therapy for cancer: current progress, unresolved questions and future directions. Angiogenesis 17: 471–494. 24482243 10.1007/s10456-014-9420-y PMC4061466

[14] Sadlecki P, Bodnar M, Grabiec M, Marszalek A, Walentowicz P, et al (2014) The role of Hypoxia-inducible factor-1 alpha, glucose transporter-1, (GLUT-1) and carbon anhydrase IX in endometrial cancer patients. Biomed Res Int 2014: 616850. 24745019 10.1155/2014/616850 PMC3972900

[15] Warburg O (1956) On the origin of cancer cells. Science 123: 309–314. 13298683 10.1126/science.123.3191.309

[16] Sedlakova O, Svastova E, Takacova M, Kopacek J, Pastorek J, et al (2014) Carbonic anhydrase IX, a hypoxia-induced catalytic component of the pH regulating machinery in tumors. Front Physiol 4: 400. 24409151 10.3389/fphys.2013.00400 PMC3884196

[17] Chinnadurai G, Vijayalingam S, Gibson SB (2008) BNIP3 subfamily BH3-only proteins: mitochondrial stress sensors in normal and pathological functions. Oncogene 27 Suppl 1 S114–127. 19641497 10.1038/onc.2009.49 PMC2925272

[18] Semenza GL (2010) Defining the role of hypoxia-inducible factor 1 in cancer biology and therapeutics. Oncogene 29: 625–634. 19946328 10.1038/onc.2009.441 PMC2969168

[19] Dodd KM, Yang J, Shen MH, Sampson JR, Tee AR (2014) mTORC1 drives HIF-1alpha and VEGF-A signalling via multiple mechanisms involving 4E-BP1, S6K1 and STAT3. Oncogene. 10.1038/onc.2014.164 PMC4172452 24931163

[20] Arsham AM, Howell JJ, Simon MC (2003) A novel hypoxia-inducible factor-independent hypoxic response regulating mammalian target of rapamycin and its targets. J Biol Chem 278: 29655–29660. 12777372 10.1074/jbc.M212770200

[21] Duvel K, Yecies JL, Menon S, Raman P, Lipovsky AI, et al (2010) Activation of a metabolic gene regulatory network downstream of mTOR complex 1. Mol Cell 39: 171–183. 20670887 10.1016/j.molcel.2010.06.022 PMC2946786

[22] Brugarolas J, Lei K, Hurley RL, Manning BD, Reiling JH, et al (2004) Regulation of mTOR function in response to hypoxia by REDD1 and the TSC1/TSC2 tumor suppressor complex. Genes Dev 18: 2893–2904. 15545625 10.1101/gad.1256804 PMC534650

[23] Wouters BG, Koritzinsky M (2008) Hypoxia signalling through mTOR and the unfolded protein response in cancer. Nat Rev Cancer 8: 851–864. 18846101 10.1038/nrc2501

[24] Rathman-Josserand M, Genty G, Lecardonnel J, Chabane S, Cousson A, et al.. (2013) Human Hair Follicle Stem/Progenitor Cells Express Hypoxia Markers. J Invest Dermatol. 10.1038/jid.2013.113 23474947

[25] Krahl D, Sellheyer K (2010) p75 Neurotrophin receptor differentiates between morphoeic basal cell carcinoma and desmoplastic trichoepithelioma: insights into the histogenesis of adnexal tumours based on embryology and hair follicle biology. Br J Dermatol 163: 138–145. 20184585 10.1111/j.1365-2133.2010.09711.x

[26] Wykoff CC, Beasley NJ, Watson PH, Turner KJ, Pastorek J, et al (2000) Hypoxia-inducible expression of tumor-associated carbonic anhydrases. Cancer Res 60: 7075–7083. 11156414

[27] Nakayama K (2009) Cellular signal transduction of the hypoxia response. J Biochem 146: 757–765. 19864435 10.1093/jb/mvp167

[28] Zhang HM, Cheung P, Yanagawa B, McManus BM, Yang DC (2003) BNips: a group of pro-apoptotic proteins in the Bcl-2 family. Apoptosis 8: 229–236. 12766483 10.1023/a:1023616620970

[29] Bowden J, Brennan PA, Umar T, Cronin A (2002) Expression of vascular endothelial growth factor in basal cell carcinoma and cutaneous squamous cell carcinoma of the head and neck. J Cutan Pathol 29: 585–589. 12453295 10.1034/j.1600-0560.2002.291003.x

[30] Weninger W, Uthman A, Pammer J, Pichler A, Ballaun C, et al (1996) Vascular endothelial growth factor production in normal epidermis and in benign and malignant epithelial skin tumors. Lab Invest 75: 647–657. 8941211

[31] Toth-Jakatics R, Jimi S, Takebayashi S, Kawamoto N (2000) Cutaneous malignant melanoma: correlation between neovascularization and peritumor accumulation of mast cells overexpressing vascular endothelial growth factor. Hum Pathol 31: 955–960. 10987256 10.1053/hupa.2000.16658

[32] Jokilehto T, Rantanen K, Luukkaa M, Heikkinen P, Grenman R, et al (2006) Overexpression and nuclear translocation of hypoxia-inducible factor prolyl hydroxylase PHD2 in head and neck squamous cell carcinoma is associated with tumor aggressiveness. Clin Cancer Res 12: 1080–1087. 16489060 10.1158/1078-0432.CCR-05-2022

[33] D'Angelo G, Duplan E, Boyer N, Vigne P, Frelin C (2003) Hypoxia up-regulates prolyl hydroxylase activity: a feedback mechanism that limits HIF-1 responses during reoxygenation. J Biol Chem 278: 38183–38187. 12876291 10.1074/jbc.M302244200

[34] Jee SH, Chiu HC, Tsai TF, Tsai WL, Liao YH, et al (2002) The phosphotidyl inositol 3-kinase/Akt signal pathway is involved in interleukin-6-mediated Mcl-1 upregulation and anti-apoptosis activity in basal cell carcinoma cells. J Invest Dermatol 119: 1121–1127. 12445202 10.1046/j.1523-1747.2002.19503.x

[35] Chen S, Nakahara T, Uchi H, Takeuchi S, Takahara M, et al (2009) Immunohistochemical analysis of the mammalian target of rapamycin signalling pathway in extramammary Paget's disease. Br J Dermatol 161: 357–363. 19438435 10.1111/j.1365-2133.2009.09179.x

[36] Chen SJ, Nakahara T, Takahara M, Kido M, Dugu L, et al (2009) Activation of the mammalian target of rapamycin signalling pathway in epidermal tumours and its correlation with cyclin-dependent kinase 2. Br J Dermatol 160: 442–445. 19016696 10.1111/j.1365-2133.2008.08903.x

[37] Karayannopoulou G, Euvrard S, Kanitakis J (2013) Differential expression of p-mTOR in cutaneous basal and squamous cell carcinomas likely explains their different response to mTOR inhibitors in organ-transplant recipients. Anticancer Res 33: 3711–3714. 24023300

[38] Chiang GG, Abraham RT (2005) Phosphorylation of mammalian target of rapamycin (mTOR) at Ser-2448 is mediated by p70S6 kinase. J Biol Chem 280: 25485–25490. 15899889 10.1074/jbc.M501707200

[39] Hardie DG (2005) New roles for the LKB1—>AMPK pathway. Curr Opin Cell Biol 17: 167–173. 15780593 10.1016/j.ceb.2005.01.006

[40] Fruman DA, Rommel C (2014) PI3K and cancer: lessons, challenges and opportunities. Nat Rev Drug Discov 13: 140–156. 24481312 10.1038/nrd4204 PMC3994981

[41] Liu LS, Colegio OR (2013) Molecularly targeted therapies for nonmelanoma skin cancers. Int J Dermatol 52: 654–665. 23679874 10.1111/ijd.12017

